# Retraction: Production of a recyclable nanobiocatalyst to synthesize quinazolinone derivatives

**DOI:** 10.1039/d6ra90007k

**Published:** 2026-01-23

**Authors:** Meenakshi Budhiraja, Bhupendra Chudasama, Amjad Ali, Vikas Tyagi

**Affiliations:** a School of Chemistry and Biochemistry, Thapar Institute of Engineering and Technology (TIET) Patiala Punjab India vikas.tyagi@thapar.edu amjadali@thapar.edu; b Center of Excellence for Emerging Materials, Thapar Institute of Engineering and Technology Patiala-147004 India; c School of Physics and Materials Science, Thapar Institute of Engineering and Technology Patiala-147004 India

## Abstract

Retraction of ‘Production of a recyclable nanobiocatalyst to synthesize quinazolinone derivatives’ by Meenakshi Budhiraja *et al.*, *RSC Adv.*, 2022, **12**, 31734–31746, https://doi.org/10.1039/D2RA04405F.

The Royal Society of Chemistry hereby wholly retracts this *RSC Advances* article due to concerns with the reliability of the data.

There are multiple repeating fragments in many of the XRD patterns in Fig. 2b.

The authors provided raw data but it does not match the published data. Experts have confirmed that the authors' response does not address the concerns.

Given the significance of these concerns, the findings presented in this paper are no longer reliable.

The authors were informed of the decision to retract this article. Meenakshi Budhiraja, Amjad Ali and Vikas Tyagi do not agree with this decision. Bhupendra Chudasama has not responded.

Laura Fisher

13th January 2026

Executive Editor, *RSC Advances*

